# Skin Wound Healing Potential and Mechanisms of the Hydroalcoholic Extract of Leaves and Oleoresin of* Copaifera langsdorffii* Desf. Kuntze in Rats

**DOI:** 10.1155/2017/6589270

**Published:** 2017-08-27

**Authors:** Lucas Fernando Sérgio Gushiken, Carlos Alberto Hussni, Jairo Kenupp Bastos, Ariane Leite Rozza, Fernando Pereira Beserra, Ana Júlia Vieira, Carlos Roberto Padovani, Marivane Lemos, Maurilio Polizello Junior, Jonas Joaquim Mangabeira da Silva, Rafael Henrique Nóbrega, Emanuel Ricardo Monteiro Martinez, Cláudia Helena Pellizzon

**Affiliations:** ^1^Department of Morphology, Institute of Biosciences of Botucatu, UNESP, Campus Botucatu, Botucatu, SP, Brazil; ^2^Department of Surgery and Veterinary Anesthesiology, School of Veterinary Medicine and Animal Science, UNESP, Campus Botucatu, Botucatu, SP, Brazil; ^3^Department of Pharmaceutical Sciences, School of Pharmaceutical Sciences of Ribeirão Preto, University of São Paulo, Campus Ribeirão Preto, Ribeirão Preto, SP, Brazil; ^4^Department of Biostatistics, Institute of Biosciences of Botucatu, UNESP, Campus Botucatu, Botucatu, SP, Brazil

## Abstract

The wound healing is a complex process which, sometimes, can be a problem in public health because of the possibility of physical disability or even death. Due to the lack of a gold standard drug in skin wound treatment and aiming at the discovery of new treatments in skin repair and the mechanisms involved in the process, we used oleoresin (OR) from* Copaifera langsdorffii* and hydroalcoholic extract of the leaves (EH) to treat rat skin wounds. For that, male* Wistar* rats were divided into groups (*n* = 8): Lanette, Collagenase, 10% EH, or 10% OR and, after anesthesia, one wound of 2 cm was made in the back of animals. The wounds were treated once a day for 3, 7, or 14 days and the wound areas were measured. The rats were euthanized and skin samples destined to biochemical, molecular, and immunohistochemical analysis. The results showed a macroscopic retraction of the wounds of 10% EH and 10% OR creams and both treatments showed anti-inflammatory activity. Molecular and immunohistochemical results demonstrated the activity of* Copaifera langsdorffii* creams in angiogenesis, reepithelialization, wound retraction, and remodeling mechanisms.

## 1. Introduction

The* Copaifera langsdorffii* Desf. Kuntze (Leguminosae), popularly known as copaiba, is a native tree from tropical regions of Latin America and Western Africa, which grows in the North, Northeast,and Central-West of Brazil, especially in the States of Amazonas, Paiva et al. [1]. Since the XVI century, the oleoresin of* Copaifera langsdorffii* has been used by Brazilian Indians to treat some diseases [2] and the popular knowledge reports the use of the plant as anti-inflammatory and antimicrobial agent and to treat skin wounds [1, 3]. Among the biological properties of copaiba, some have already been studied such as anti-inflammatory agent [4], antinociceptive [5], antioxidant [6], gastrointestinal diseases [6–9], pulmonary and urinary disorders [10], urolithiasis [11], and analgesic agent [1, 12]. Furthermore, there are some works [1, 13–17] which studied the wound healing potential of* Copaifera langsdorffii* through macroscopic analysis and collagen synthesis mechanisms, but there is the necessity of more researches about the wound healing mechanisms of the plant. Most of the studies from* Copaifera* genus used the oleoresin, with few studies using the aerial parts [18]. However, the aromatic and phenolic compounds presented in aerial parts of copaiba are important in cosmetic and pharmaceutical industries [19], besides the use of hydroalcoholic extract from the leaves to treat urolithiasis [11].

Skin wound healing is a physiological process that depends on molecular and cellular mechanisms and can be didactically divided into 3 overlapping and interdependent phases: inflammatory, proliferative, and remodeling phases [20]. Nonhealing wounds have a significant impact in public health and in the expenditure of public resources because the wounds can cause physical and psychological deficiency, or even death [21]. Although there are several treatment options on the market for skin wounds, many of them have high costs to the patient, due to the long duration of treatment [22]. Because of this, industry and academic researchers are exploring new therapeutic strategies to accelerate wound closure, including the use of plants and natural products [23]. For this reason, our study analyzed the wound healing activity of topical formulations containing oleoresin (OR) and hydroalcoholic extract of leaves (EH) from* Copaifera langsdorffii* in rat skin, with comprehension of the main process involved in the treatments of the 3 different phases of wound healing and analysis of the pathways, in order to understand the mechanisms of a possible natural drug in the treatment of skin wound healing.

## 2. Material and Methods

### 2.1. Plant Material

90 mL of the* Copaifera langsdorffii* oleoresin was obtained from a specimen located in Cajurú, São Paulo, Brazil. A voucher specimen (SPFR 10120) identified by Milton Groppo Junior was deposited in the Biology Department, FFCLRP, University of São Paulo (USP).* Copaifera langsdorffii* leaves were collected in Ribeirão Preto, São Paulo, Brazil, and identified by Milton Groppo Junior. A voucher specimen (SPFR 10120) was deposited in the Biology Department, FFCLRP, University of São Paulo (USP).

### 2.2. Phytochemical Characterization of* Copaifera langsdorffii*

The volatile sesquiterpenes of* Copaifera langsdorffii* oleoresin was analyzed by using the gas chromatography linked mass spectrometer (GC-MS) as described by Souza et al. (2011) [24]. Hydroalcoholic extract of the copaiba leaves was analyzed by HPLC-MS as described by Alves et al. (2013) [18].

### 2.3. Composition and Stability Tests of* Copaifera langsdorffii* Creams

The vehicle (topical formulation) was comprised of an aqueous phase composed of 75.8% water and 4.0% propylene glycol and an organic phase composed of 17% Lanette cream, 3% hard paraffin, 0.15% Nipagin, and 0.05% Nipasol, pH 6.86 ± 0.0215. For that, the organic phase was heated till melting, and the aqueous phase was heated at 35°C. Then, the two phases were combined and stirred till they cooled dawn to room temperature. All the treatments used in the skin wound healing model (Lanette; Collagenase; 1%, 5%, and 10% EH; and 1%, 5%, and 10% OR) were made using the vehicle formulation.

In order to verify the stability, the creams were submitted to stability tests according to the parameters and rules of the National Health Surveillance Agency (ANVISA). All creams were subjected to centrifugation at 3000 rpm for 30 minutes to verify the possible separation of phases. They were stored under three different temperatures (4 ± 2°C, 27 ± 2°C, and 40 ± 2°C) in an incubator, with 5% humidity. After 1, 7, 15, 30, 60, and 90 days, the samples were collected for physical and chemical stability analysis. To evaluate the physical stability, visual analysis was used in which the formulations were observed for color alteration, phase separation, and homogeneity. The alterations in pH values were verified. The samples were diluted in methanol-water (1 : 1) solution, and the pH value was determined in ambient temperature (27 ± 2°C) with a pHmeter. To analyze the chemical stability, 1 g of each formulation was weighed and dissolved in 1 mL of methanol HPLC grade and 1 mL of hexane HPLC grade was added for the clean-up process. Before chromatographic analysis, each fraction was dissolved to a concentration of 1 mg/mL. The OR formulations were analyzed using a GC-FID (gas chromatography with flame ionization detector) system and the EH formulations were analyzed using HPLC-MS, according to the methods described by Sousa et al. (2011) [25] and Motta et al. (2017) [8].

### 2.4. Animals and Wound Healing Activity

#### 2.4.1. Animals

The assay was performed on male* Wistar* albino rats* (Rattus norvegicus)* weighing 250 ± 30 g, supplied by the Central Animal House, UNESP, Botucatu. The rats were acclimated in individual cages for 1 week, in a controlled environment (23 ± 2°C, 12 hours' dark-light cycle, food and water ad libitum). After the acclimatization period, the animals were subjected to the experimental procedures. All experiments involving animals were performed according to the experimental protocols (Protocol 413/2012) approved by the Ethics Committee on Animal Use (CEUA/IBB/UNESP).

#### 2.4.2. Excision Wounds and Treatments

The animals were anesthetized with intraperitoneal ketamine (80 mg/kg) and xylazine (10 mg/kg). After anesthesia, the animal's back was shaved, and a lesion was made in the dorsal region with a 2 cm diameter punch. Subsequently, the animals were monitored to total recovery and randomly distributed into 8 groups (*n* = 8/group): Lanette (vehicle), Collagenase 1.2 IU (reference drug), 1% OR, 5% OR, and 10% OR, and 1% EH, 5% EH, and 10% EH creams. Immediately after the surgical excision, the wounds were topically treated for 3 different experimental periods: 3, 7, or 14 days (*n* = 64 animals/period). The treatments were administered every day, once a day during each period. To establish wound retraction, the lesion size was marked daily in plastic films. After each period of treatment (3, 7, and 14 days), the animals were euthanized, and their wounds were collected for biochemical, molecular, and histological analysis.

### 2.5. Macroscopic Analysis

The wound retraction was analyzed daily in each treatment period group. The measures were submitted to planimetry analysis using digital pachymeter. The area of wound retraction was calculated (%) by the following formula:

%  wound  retraction = {(initial  area  of  the  wound − area  of  wound  measured)/initial  area  of  the  wound}*∗*100

The data of the wound areas were expressed as the mean ± standard deviation.

### 2.6. Biochemical Analysis

After the macroscopic analysis, we selected 10% OR and 10% EH—single concentration with effect on macroscopic assay—to proceed with the biochemical assay. The skin samples were processed and the levels of cytokines TNF-*α*, IL-1*β*, IL-6, and IL-10 were quantified through the protocol of ELISA kit R&D Systems, Minneapolis, USA. The data of cytokines were calculated according to protein amount of the sample (pg/mg of protein) [26].

### 2.7. Reverse Transcription-qPCR (RT-qPCR) Analysis

After the macroscopic analysis, we selected 10% OR and 10% EH—single concentration with effect on macroscopic assay—to proceed with the molecular assay. The RNA of the skin samples was extracted using the phenol/chloroform method. After the extraction, the RNA was treated with DNase I (Thermo Fischer Scientific, USA) to remove possible contamination with genomic DNA. The DNA synthesis was made by reverse transcription using the kit SuperScript II according to the proceedings described in the kit (Thermo Fischer Scientific, USA). To analyze the relative mRNA expression levels of* COL1A1*,* COL3A1*,* EGF*,* FGF-2,* and* TGF-β1* (Table 1) between our treatments, the RT-qPCR method was used as previously described [27, 28].

### 2.8. Microscopic Analysis

After the macroscopic analysis, we selected 10% OR and 10% EH—single concentration with effect on macroscopic assay—to proceed the microscopic assay. The samples of each animal of groups Lanette, Collagenase, 10% EH, and 10% OR were fixed with 80% alcohol/acetic acid/formalin (8 : 1 : 1) and processed in paraffin.

For hematoxylin & eosin (HE) staining, the number of vessels of all slices (each slice representing the respective rat) was analyzed. Three different regions of the skin were analyzed for all slices: normal skin (skin without wound) and border and center of the wounds. 15 photomicrographs of each slice were analyzed with 40x lens and the software CellSens Standard (Olympus, USA), being 5 photomicrographs from normal skin, 5 from the border, and 5 from the center of the wounds. The number of blood vessels was obtained by counting of vessels present in the photomicrographs using a hand counter.

The immunohistochemical assay was made with antibodies collagen 1 (1 : 100 *μ*L), collagen 3 (1 : 100 *μ*L), EGF (1 : 200 *μ*L), FGF-2 (1 : 500 *μ*L), MIF (1 : 300 *μ*L), MMP-2 (1 : 100 *μ*L), MMP-9 (1 : 100 *μ*L), PCNA (1 : 200 *μ*L), TGF-*β*1 (1 : 300 *μ*L), and VEGF (1 : 100 *μ*L). The skin samples were fixed with 80% alcohol/acetic acid/formalin (8 : 1 : 1) and processed in paraffin. Subsequently, slices of 5 *μ*m were prepared and submitted to antigen retrieval by pressure (20 psi/125°C). 15 photomicrographs of each slice were analyzed with 40x lens and the software CellSens Standard (Olympus, USA), being 5 photomicrographs from normal skin, 5 from the border, and 5 from the center of the wounds. The immunolabeled area was quantified totalizing 100000 *μ*m^2^/slice. The quantification was made with the software AVSoftBioView.

### 2.9. Statistical Analysis

For statistical analysis, all of the data were determined by analysis of variance of the repeated measures in the independent groups followed by the Newman-Keuls multiple comparison test ± standard deviation, considering a 5% level of significance. The analyses were performed using GraphPad Prism version 5.01 software.

## 3. Results

### 3.1. Stability Test of* Copaifera langsdorffii* Creams

**Figure 1:**
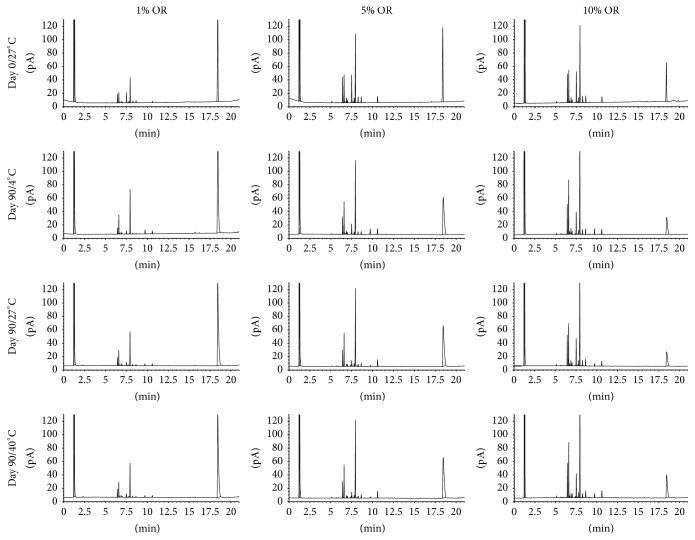
Chromatograms obtained by GC-FID of* Copaifera langsdorffii *oleoresins.

**Figure 2:**
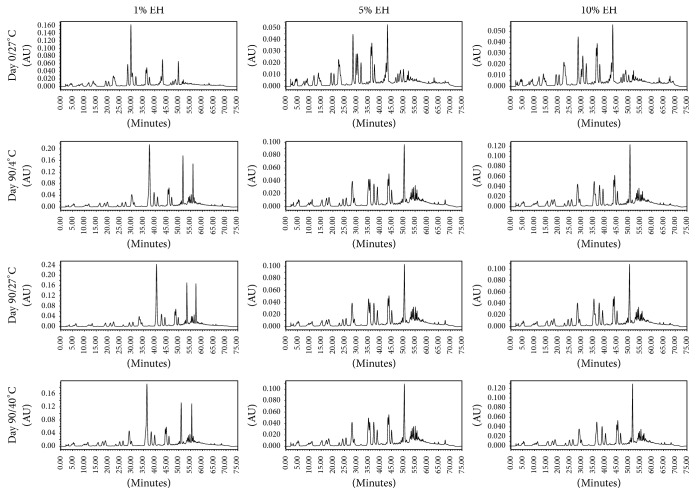
Chromatograms obtained by HPLC-MS of* Copaifera langsdorffii *hydroalcoholic extract of the leaves.

In the centrifugation preliminary test, it was verified that the samples were stable, with no phase separation, allowing the continuation in the physical and chemical tests. During the 90 days of the test, the creams did not show any change in the physical stability, like visual characteristics, alterations in color, and formation of phase, which demonstrates homogeneity. There was no significant difference between the pH values of the creams at the different storage durations (Table 2). In the analysis of the OR creams by GC-FID, there were no alterations in chromatographic profile after 90 days of test in 4°C and 27°C and the major compounds are present in all chromatograms. However, the samples stored for 90 days at 40 ± 2°C showed a different profile, suggesting there is a degradation of the compounds (Figure 1). The HPLC-MS profile of the EH creams at 4°C and 27°C did not show any difference, with all formulations remaining stable after 90 days. However, as well as the OR creams, EH samples stored for 90 days at 40 ± 2°C showed an alteration of chemical profile, suggesting the degradation of their compounds (Figure 2).

### 3.2. Macroscopic Analysis

**Figure 3:**
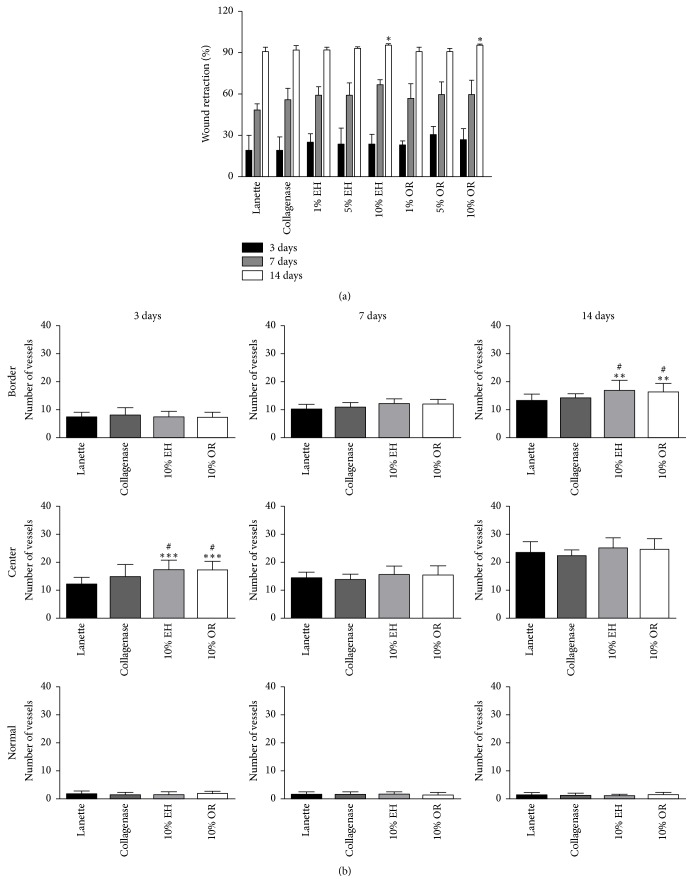
(a) Skin wound closure (%) of rats submitted to treatments (*n* = 8) Lanette, Collagenase, EH, or OR 1%, 5%, and 10% for 3, 7, and 14 days. Number of vessels in HE staining of rat skin wounds treated with Lanette, Collagenase, 10% EH, or 10% OR for 3, 7, and 14 days. ^*∗*^*p* < 0.05, ^*∗∗*^*p* < 0.01, and ^*∗∗∗*^*p* < 0.001, compared with Lanette group. ^#^*p* < 0.05, compared with Collagenase group, using ANOVA followed by Newman-Keuls test.

In this study, the animals treated with Lanette, Collagenase, 1%, 5%, and 10% EH, or OR during the first 3 and 7 days did not show significant differences between the groups in the wound area. After 14 days of treatment, a significant difference was observed in wound closure after treatment with 10% EH (95.1% wound reduction) and 10% OR (94.72% wound reduction), compared with those treated with Lanette cream (89.75 wound reduction) (Figure 3(a)). According to the macroscopic results, the groups of 10% EH and 10% OR were selected to perform the biochemical, molecular, and histological analysis.

### 3.3. ELISA Biochemical Analysis

**Figure 4:**
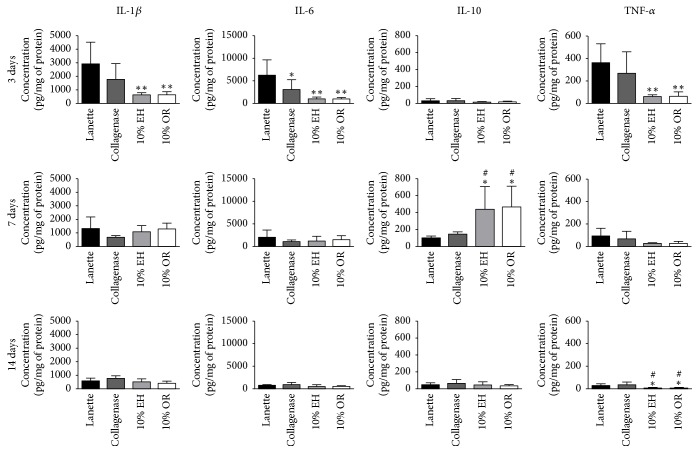
Concentration of TNF-*α*, IL-1*β*, IL-6, and IL-10 (pg/mg of protein) in skin wound of rats treated with Lanette, Collagenase, 10% EH, or 10% for 3, 7, and 14 days. ^*∗*^*p* < 0.05 and ^*∗∗*^*p* < 0.01, compared with Lanette. ^#^*p* < 0.05, compared with Collagenase, using ANOVA followed by Newman-Keuls test (*n* = 8).

The results of ELISA assay demonstrated the anti-inflammatory effect of 10% EH and 10% OR, reducing the concentrations of proinflammatory cytokines TNF-*α*, IL-1*β*, and IL-6 after 3 days of treatment and increasing the anti-inflammatory cytokine IL-10 after 7 days, compared with Lanette cream (Figure 4).

### 3.4. RT-qPCR Gene Expression

**Figure 5:**
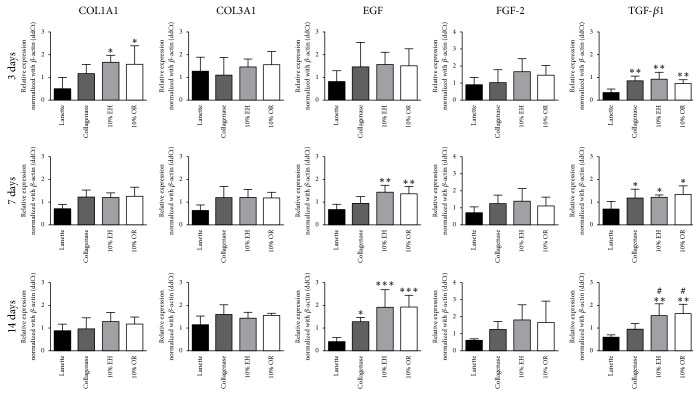
Gene expression by RT-qPCR of* COL1A1*,* COL3A1*,* EGF*,* FGF-2,* and* TGF-β1* in skin wounds treated for 3, 7, and 14 days with Lanette, Collagenase, 10% EH, or 10% OR. The gene expression was normalized according to*β-ACTIN* gene. ^*∗*^*p* < 0.05, ^*∗∗*^*p* < 0.01, and ^*∗∗∗*^*p* < 0.001, compared with Lanette. ^#^*p* < 0.05, compared with Collagenase, using ANOVA followed by Newman-Keuls test (*n* = 8).

The molecular data of relative gene expression of collagen 1-alpha-1, collagen 3-alpha-1, EGF, FGF-2, and TGF-*β*1 can be observed in Figure 5.

There was an increase in the expression of collagen 1-alpha-1 in 10% EH and 10% OR compared to Lanette treatment after 3 days, but no difference was observed after 7 and 14 days. There were no significant changes in the expression of collagen 3-alpha-1 and FGF-2 in any periods studied. The relative expression of EGF was increased in groups 10% EH and 10% OR after 7 days compared with Lanette, and after 14 days the treatments Collagenase, 10% EH, and 10% OR showed enhancement of EGF expression compared with Lanette. The expression of TGF-*β*1 was increased after 3 and 7 days in groups Collagenase, 10% EH, and 10% OR compared with Lanette and, after 14 days, 10% EH and 10% OR showed the increase of TGF-*β*1 expression compared to Lanette and Collagenase treatments.

### 3.5. Microscopic Analysis

The HE staining allowed the counting of vessels in the normal skin and border and center of the wounds. There was an increase in the number of vessels in groups 10% EH and 10% OR at center of the wounds compared to Lanette and Collagenase groups after 3 days and in the border of wounds after 14 days. There was no vascular alteration in normal skin of tested groups in any period studied (Figure 3(b)).

**Figure 6:**
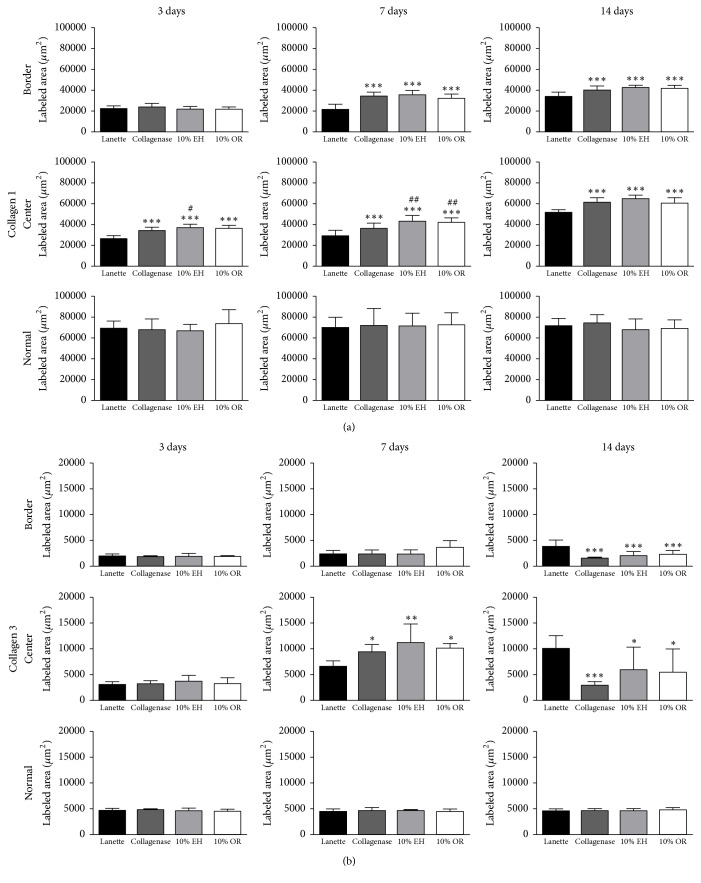
Immunolabeled area (*μ*m^2^) for collagen 1 and collagen 3 of the three regions from skin wounds treated with Lanette, Collagenase, 10% EH, or 10% OR for 3, 7, and 14 days. ^*∗*^*p* < 0.05, ^*∗∗*^*p* < 0.01, and ^*∗∗∗*^*p* < 0.001, compared with Lanette. ^#^*p* < 0.05 and ^##^*p* < 0.01, compared with Collagenase, using ANOVA followed by Newman-Keuls test (*n* = 8).

**Figure 7:**
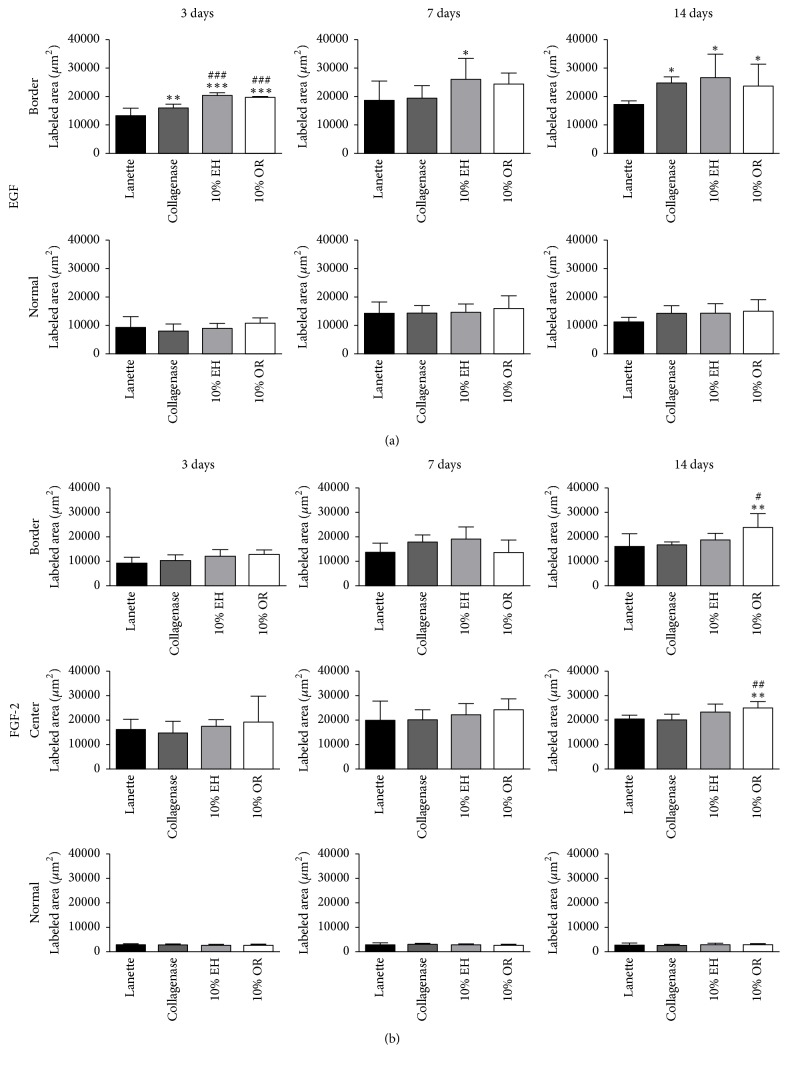
Immunolabeled area (*μ*m^2^) for EGF and FGF-2 of the three regions from skin wounds treated with Lanette, Collagenase, 10% EH, or 10% OR for 3, 7, and 14 days. ^*∗*^*p* < 0.05, ^*∗∗*^*p* < 0.01, and ^*∗∗∗*^*p* < 0.001, compared with Lanette. ^#^*p* < 0.05, ^##^*p* < 0.01, and ^###^*p* < 0.001, compared with Collagenase, using ANOVA followed by Newman-Keuls test (*n* = 8).

**Figure 8:**
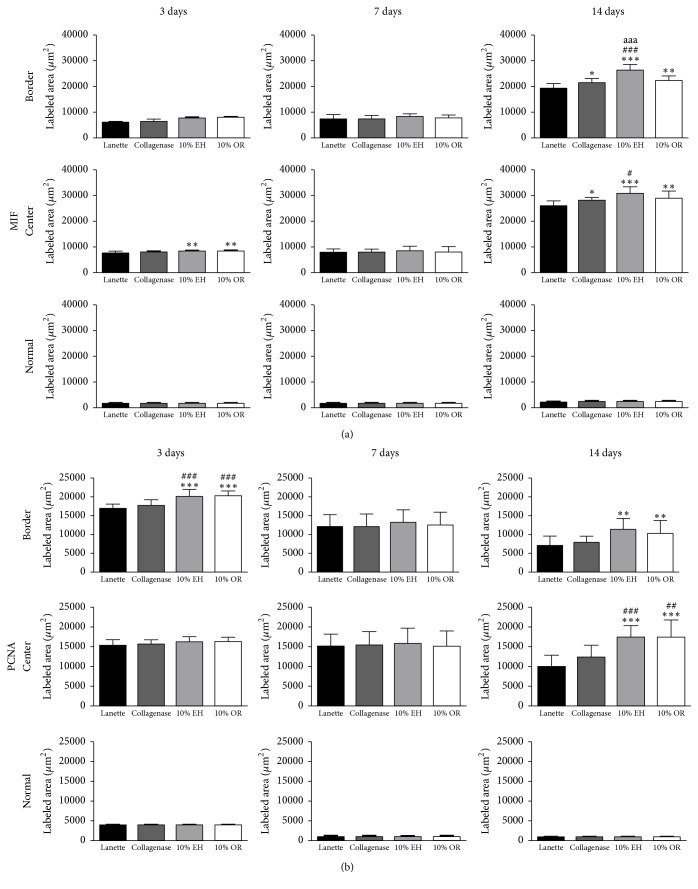
Immunolabeled area (*μ*m^2^) for MIF and PCNA of the three regions from skin wounds treated with Lanette, Collagenase, 10% EH, or 10% OR for 3, 7, and 14 days. ^*∗*^*p* < 0.05, ^*∗∗*^*p* < 0.01, and ^*∗∗∗*^*p* < 0.001, compared with Lanette. ^#^*p* < 0.05, ^##^*p* < 0.01, and ^###^*p* < 0.001, compared with Collagenase. ^aaa^*p* < 0.001, compared with 10% OR, using ANOVA followed by Newman-Keuls test (*n* = 8).

**Figure 9:**
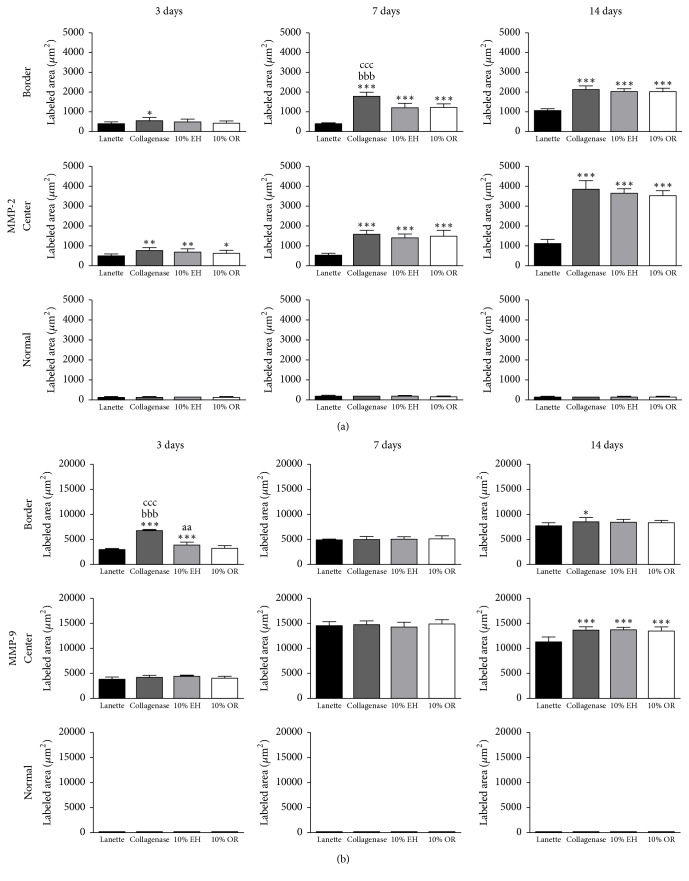
Immunolabeled area (*μ*m^2^) for MMP-2 and MMP-9 of the three regions from skin wounds treated with Lanette, Collagenase, 10% EH, or 10% OR for 3, 7, and 14 days. ^*∗*^*p* < 0.05, ^*∗∗*^*p* < 0.01, and ^*∗∗∗*^*p* < 0.001, compared with Lanette. ^aa^*p* < 0.01, compared with 10% OR. ^bbb^*p* < 0.001, compared with 10% EH. ^ccc^*p* < 0.001, compared with 10% OR, using ANOVA followed by Newman-Keuls test (*n* = 8).

**Figure 10:**
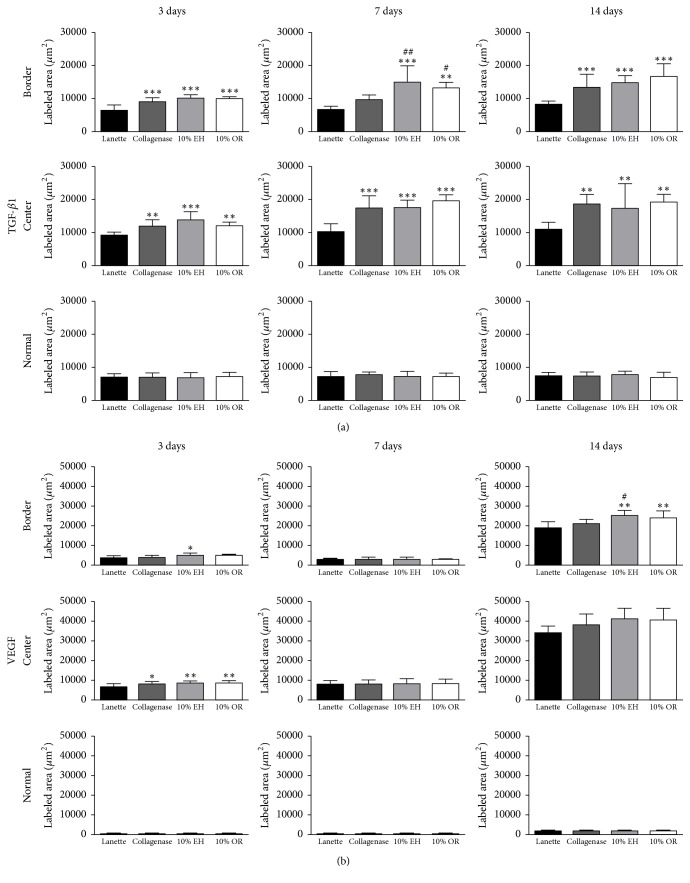
Immunolabeled area (*μ*m^2^) for TGF-*β*1 and VEGF of the three regions from skin wounds treated with Lanette, Collagenase, 10% EH, or 10% OR for 3, 7, and 14 days. ^*∗*^*p* < 0.05, ^*∗∗*^*p* < 0.01, and ^*∗∗∗*^*p* < 0.001, compared with Lanette. ^#^*p* < 0.05 and ^##^*p* < 0.01, compared with Collagenase, using ANOVA followed by Newman-Keuls test (*n* = 8).

The immunohistochemistry of collagen 1 showed the increase of immunolabeling of Collagenase, 10% EH, and 10% OR in wound border after 7 and 14 days compared to Lanette and enhancement of collagen 1 in the center of wounds of the same groups in all periods studied (Figure 6(a)). About the immunolabeling of collagen 3, there was an increase of this protein in Collagenase, 10% EH, and 10% OR in the center of wounds after 7 days. In 14 days, the level of immunolabeling of collagen 3 was decreased in the center and border of wounds in the three groups compared to Lanette (Figure 6(b)). The immunolabeling to EGF showed the increase of labeling area in the border of groups Collagenase, 10% EH, and 10% OR after 3 and 14 days (Figure 7(a)). The immunohistochemistry of FGF-2 showed that this protein was enhanced only in the group 10% OR after 14 days, compared to Lanette and Collagenase treatments (Figure 7(b)). With regard to MIF, the immunolabeling was increased in creams of copaiba after 3 days and the highest labeling of groups Collagenase, 10% EH, and 10% OR after 14 days, with 10% EH demonstrating higher labeling than Collagenase and 10% OR (Figure 8(a)). The results of MMP-2 and MMP-9 showed the higher labeling of these proteins in the border and center of Collagenase, 10% EH, and 10% OR treated groups especially after 14 days, besides the important activity of these enzymes presented in Collagenase treatment in 3 and 7 days (Figure 9). The immunohistochemical data of PCNA demonstrated the increase of labeled cells of groups 10% EH and 10% OR in the border of wounds after 3 and 14 days and in the center of wounds after 14 days (Figure 8(b)). The immunohistochemistry of TGF-*β*1 showed an increase of the labeled area in the center and border of wounds from groups Collagenase, 10% EH, and 10% OR compared to Lanette after 3, 7, and 14 days (Figure 10(a)). The immunohistochemical results of VEGF showed the increase of labeling of Collagenase, 10% EH, and 10% OR in the center of wounds after 3 days and higher labeling of 10% EH and 10% OR in the border of wounds after 14 days (Figure 10(b)).

## 4. Discussion

As well as other plants whose wound healing potential derived from popular knowledge has been proved experimentally [29–33], this article proved the effectiveness of* Copaifera langsdorffii* creams in rat skin wound healing, according to macroscopic retraction of wounds after 14 days, besides the effect of leaves extract and oleoresin in the anti-inflammatory activity, boost in reepithelialization, angiogenesis, cell proliferation, and extracellular matrix remodeling. By this way, there is the possibility of using OR and EH in skin wound healing.

One of the major challenges in product development is to ensure the stability of pharmaceutical formulations for long periods without degrading their constituents by processes such as oxidation and hydrolysis [34, 35]. To ensure the stability of the formulations, physical and chemical tests were made in 3 different temperatures. According to the chromatographic profile and comparing the data present in the literature [8, 25, 36], the data indicate that the formulations are stable up to 90 days at temperatures of 4 and 27°C, while the formulations submitted to 40°C appear to be degraded, observing the chromatographic profile obtained by GC-FID or by HPLC-MS.

After synthesis of the hemostatic clot at the wound site, platelets and cells from the region secrete cytokines, such as IL-1*β*, IL-6, and TNF-*α*, which stimulate leukocyte infiltration into the region, initiating the inflammatory response [37]. Studies indicate that both proinflammatory factors such as TNF-*α*, IL-1*β*, and IL-6 and anti-inflammatory factors such as IL-10 are involved in mechanisms that comprise the three phases of wound healing process [30, 38–42]. However, the inflammatory response must occur rapidly for the correct healing of the lesions, because the continuous secretion of cytokines and the debridement of the wound by leukocyte lead to chronic inflammation, delaying the healing mechanism, with possible loss of tissue function. Thus, the control of pro- and anti-inflammatory molecules is necessary for the physiological healing mechanism, with studies demonstrating the use of plants to decrease inflammation of wounds [43–47]. Based on the ELISA results, 10% EH and 10% OR creams showed anti-inflammatory effect, reducing concentrations of proinflammatory cytokines (TNF-*α*, IL-1*β*, and IL-6) and increasing the level of the anti-inflammatory cytokine IL-10, inhibiting chronic inflammation which avoids tissue fibrosis formation and delayed healing of lesions.

Together with hemostatic clot formation and inflammation, the cells of the region synthesize growth factors which stimulate cells migration and proliferation and the synthesis of provisional extracellular matrix responsible for the local filling and maintenance of healing mechanisms until the remodeling of the permanent extracellular matrix [48]. FGF-2 is a growth factor responsible for the migration and proliferation of fibroblasts and endothelial cells at the site of injury. Although the molecular results showed no differences in FGF-2 gene expression between the treatments, the increase of this growth factor in the immunohistochemical analysis was observed for the group 10% OR, a result that can be explained by the posttranscriptional regulation, which changes the protein synthesis with no effects on gene expression, such as the multilevel control of FGF-2 protein synthesis controlled by 3′-UTR of its mRNA [49]. Thus, 10% OR treatment increased FGF-2 levels, stimulating the fibroblasts proliferation and migration. Other growth factors were released during the skin wound healing, and the TGF-*β*1 is one of the healing mediators which has the largest range of activities, participating in all healing phases [50]. This protein acts on the migration and proliferation of cells from the injured region, participating in angiogenesis, reepithelialization, and synthesis of granulation tissue and collagen remodeling [51]. By this way, the increase of TGF-*β*1 in the molecular and immunohistochemical analyses in* Copaifera langsdorffii* treatments, together with immunohistochemistry for the cell proliferating marker PCNA, demonstrates the effect of TGF-*β*1 on proliferation of cells in the lesion.

The reconstruction of the vascular network of the injury is essential to the wound healing. With mediators secreted by the cells of the region, such as angiopoietins, VEGF, and TGF-*β*1, there is the stimulus for proliferation, migration, and structuring of endothelial cells [52]. Therefore, the results obtained by counting of vessels and VEGF immunolabeling indicate the stimulation of the treatments 10% EH and 10% OR in the formation of vessels. Furthermore, the TGF-*β*1 also can affect angiogenesis, acting on the migration and structuring of endothelial cells by supraregulation of integrin receptors [53].

There is another essential mechanism for the skin wound healing; the reepithelialization begins some hours after the injury, but it has the most evident activity in the proliferative phase of healing, finishing during the remodeling of the extracellular matrix [48]. The keratinocytes present at the border of the wounds secrete growth factors that stimulate the proliferation and migration of these cells to the covering of the injured area. One of these factors, EGF, is a molecule that exhibits mitogenic and migratory activity on the keratinocytes of the wound border [54]. To recover the wounds by keratinocyte migration, extracellular matrix metalloproteinases 2 and 9 (MMP-2 and MMP-9) dissolve adhesion molecules between keratinocytes and keratinocytes and basal membrane [55]. Therefore, the gene expression and immunolabeling results of EGF, MMP-2, and MMP-9 demonstrated the influence of* Copaifera langsdorffii* creams stimulating the wound reepithelialization mechanism.

The last stage of skin wound healing is the remodeling phase, in which the provisional extracellular matrix is remodeled, the injured area is completely reepithelialized, and a myofibroblast-mediated contractile response of the injury occurs. Because of their multiple binding sites with collagen, myofibroblasts bind to collagen fibers and contract, reducing the wound area [56], and macrophage migration inhibitory factor (MIF) is a highly expressed protein in the skin, with functions in the inflammatory phase of healing and differentiation of fibroblast to myofibroblasts [57, 58]. The myofibroblasts differentiation is essential to skin wound healing, avoiding the delay in the closure of the lesions and tissue fibrosis [59]. From the TGF-*β*1 stimulus, phenotypic alterations of the fibroblasts present at the wound border begin, which synthesize *α*-smooth muscle actin, cell adhesion receptors, and other contractile proteins, characterizing the differentiation for myofibroblasts [60, 61]. The results obtained with MIF and TGF-*β*1 tests showed that* Copaifera langsdorffii *creams affect myofibroblast differentiation through these pathways, with possibility of myofibroblast retraction being responsible for wound area reduction.

In addition to myofibroblasts-mediated wound retraction, the remodeling phase is characterized by maturation of the extracellular matrix. The main mediators responsible for the degradation of collagen 3 in this phase are metalloproteinases 1 and 8 [55], while the synthesis of collagen 1 occurs by TGF-*β*1 stimulus [37]. The results of the molecular and immunohistochemical analyses for collagen 1 and collagen 3 demonstrate the influence of Collagenase, 10% EH, and 10% OR treatments on the synthesis of these proteins, with one of the mechanisms involved maybe related to the increased activity of TGF-*β*1, stimulating the synthesis of collagen 3 in the initial stages of healing and remodeling of collagen 1 in the final phase. Moreover, the molecular and immunohistochemical results of collagen 3 can be changed by posttranscriptional regulation of collagen 3 protein [62, 63].

## 5. Conclusions

The results presented showed the wound healing activity of the formulations based on hydroalcoholic extract of the leaves and oleoresin of* Copaifera langsdorffii* in the concentration of 10%, both formulations decreasing the wound area compared to the control group. In addition, although there are similarities between the mechanisms of the formulations 10% EH and 10% OR, characterized by anti-inflammatory activity, angiogenesis stimulation, reepithelialization, wound retraction, and extracellular matrix remodeling (Table 3), there are some differences between their mechanisms, such as the higher expression of FGF-2 in the oleoresin group, characterizing its influence on the fibroblast proliferation pathway and collagen synthesis, and the higher MIF expression in the leaves extract, stimulating the myofibroblast differentiation in the retraction of the lesion area.

## Figures and Tables

**Table 1:** Primers used in RT-qPCR.

Gene	Primers sequence	Product	Melting temperature	Access number^*∗*^
*β-ACTIN*	F: 5′-CCCTGGCTCCTAGCACCAT-3′	80 bp	60°C	NM_031144. 3
	R: 5′-GATAGAGCCACCAATCCACACA-3′			
*COL1A1*	F: 5′-CATGTTCAGCTTTGTGGACCT-3′	94 bp	60°C	NM_053304. 1
	R: 5′-GCAGCTGACTTCAGGGATGT-3′			
*COL3A1*	F: 5′-GGGATCCAATGAGGGAGAAT-3′	128 bp	60°C	NM_032085. 1
	R: 5′-CCTTGCGTGTTTGATATT-3′			
*EGF*	F: 5′-CTCAGGCCTCTGACTCCGAA-3′	93 bp	60°C	NM_012842. 1
	R: 5′-ATGCCGACGAGTCTGAGTTG-3′			
*FGF-2*	F: 5′-GATCCCAAGCGGCTCTACTG-3′	105 bp	60°C	NM_019305. 2
	R: 5′-TAGTTTGACGTGTGGGTCGC-3′			
*TGF-β1*	F: 5′-GGGCTACCATGCCAACTTCTG-3′	82 bp	60°C	NM_021578. 2
	R: 5′-GAGGGCAAGGACCTTGCTGTA-3′			

F (forward) and R (reverse). ^*∗*^National Center for Biotechnology Information, Nucleotide.

**Table 2:** pH alterations of the formulations containing EH or OR in different temperatures after 0, 1, 7, 15, 30, 60, and 90 days.

Temperature (°C)	Lanette	OR			EH		
		1%	5%	10%	1%	5%	10%
4 ± 2°C	6,86 ± 0,02	6,59 ± 0,03	6,78 ± 0,01	6,62 ± 0,03	6,59 ± 0,03	6,53 ± 0,02	6,58 ± 0,03
27 ± 2°C	6,74 ± 0,02	6,35 ± 0,03	6,82 ± 0,02	6,60 ± 0,03	6,83 ± 0,03	6,77 ± 0,02	6,69 ± 0,03
40 ± 2°C	6,76 ± 0,02	6,75 ± 0,02	6,78 ± 0,03	6,48 ± 0,03	6,47 ± 0,03	6,64 ± 0,03	6,57 ± 0,03

**Table 3:** Comparison of the mechanisms involved in skin wound healing between groups Collagenase, 10% EH, and 10% OR.

Mechanism	Groups		
	Collagenase	10% EH	10% OR
Inflammation	—	↓	↓
Cell proliferation	—	↑	↑
Angiogenesis	—	↑	↑
Reepithelialization	↑	↑	↑
Wound retraction	—	↑	↑
Extracellular matrix remodeling	↑	↑	↑

↑: increasing compared to Lanette; ↓: decreasing compared to Lanette; —: not effective.
